# A public health perspective on virtual reality interventions: exploring the impact of VR extreme sports on stress, anxiety, and depression in men with social anxiety disorder

**DOI:** 10.3389/fpubh.2025.1617483

**Published:** 2025-06-24

**Authors:** Li Wang, Hossein Faridniya, Haiyang Yu

**Affiliations:** ^1^Department of Athletic Training, Hebei Sport University, Hebei, China; ^2^Sport Management, Faculty of Farabi, University of Tehran, Qom, Iran; ^3^Sports Institute, Nanchang Institute of Technology, Jiangxi, China

**Keywords:** men anxiety, men health, virtual reality training, exercise therapy, social anxiety

## Abstract

**Objective:**

Men diagnosed with social anxiety disorder (SAD) often face significant challenges in daily functioning, particularly within social settings. This study aimed to evaluate the effectiveness of virtual reality (VR)-based extreme sports games in reducing symptoms of depression, anxiety, and stress in this population. The intervention was designed to offer an engaging, immersive, and potentially less stigmatizing alternative to conventional therapeutic approaches.

**Methods:**

A quasi-experimental design with pre-test and post-test assessments was employed. Eighty-four men with SAD were selected through convenience sampling and randomly assigned to either an experimental group (*n* = 42) or a control group (*n* = 42). The experimental group participated in VR extreme sports sessions. Psychological symptoms were measured using the Depression, Anxiety, and Stress Scale (DASS-21), and data were analyzed via Analysis of Covariance (ANCOVA). Normality of data distribution was confirmed using the Kolmogorov–Smirnov test (*p* > 0.05), and Levene’s test also confirmed the homogeneity of variances (*p* > 0.05), supporting the use of ANCOVA to compare adjusted post-test scores between groups.

**Results:**

The VR-based intervention led to significant reductions across all three measured domains. Depression (*η*^2^ = 0.916), anxiety (*η*^2^ = 0.901), and stress (*η*^2^ = 0.829) levels showed substantial improvement in the experimental group compared to the control group.

**Conclusion:**

These findings highlight the promise of VR-based extreme sports as a novel, non-pharmacological intervention for men with SAD. By enabling controlled exposure to anxiety-provoking situations within a safe and immersive environment, the intervention effectively alleviated symptoms of depression, anxiety, and stress. Moreover, this approach may overcome common treatment barriers such as stigma and reluctance toward traditional therapy. Future large-scale, longitudinal studies are recommended to validate these outcomes and explore their long-term sustainability.

## 1 Introduction

Social anxiety is one of the most common psychological disorders affecting a large number of individuals. This disorder is typically characterized by an intense and irrational fear of social situations, leading individuals to avoid social interactions (1). Men suffering from social anxiety experience significant challenges in various aspects of their daily lives, including social, occupational, and educational relationships (2). Such challenges often lead to heightened stress, emotional tension, and anxiety in routine activities, impacting not only themselves but also weakening family stability and spreading anxiety and self-doubt among other family members (3). Traditional therapeutic approaches for this disorder generally include cognitive-behavioral therapy (CBT) and pharmacotherapy; however, these methods may not be effective for all patients and often carry multiple side effects (4). It has also been reported that men with this disorder sometimes resist pharmacological treatments and are reluctant to seek help from a therapist (5). Consequently, non-pharmacological interventions leveraging sports activities have been recommended to reduce daily anxiety and enhance self-confidence (6). One such intervention is through extreme sports. Extreme sports like paragliding, rock climbing, and skydiving are generally associated with high excitement and physical risks, often inducing elevated levels of stress and anxiety in participants (7). Due to their high excitement and stress-inducing nature, these sports could serve as a potential tool for confronting social anxiety within controlled virtual reality environments (8).

To date, numerous studies have examined the effects of physical exercise and extreme sports on enhancing self-confidence and reducing anxiety. For instance, Pustovojt demonstrated that high-adrenaline sports impact not only the body’s physiology but also individuals’ psychological states (9). Athletes involved in these activities tend to develop greater emotional and psychological resilience to stress. Additionally, Lochbaum et al. in their meta-analysis review, found that self-confidence is a key factor for improving athletic performance in men, with individual and challenging sports exerting a greater influence on boosting self-confidence than team sports (10). Similarly, Sheng Yen Lee reported that men participating in extreme sports such as rock climbing and high-altitude activities often experience improved self-confidence and reduced anxiety (11). Other studies, including those by Makar et al. (12), Lin Wang et al. (13), and Eather (14), have further highlighted the positive effects of sports and extreme sports on alleviating daily psychological stress and enhancing levels of stress and anxiety management in individuals’ daily lives.

However, an aspect that has received limited attention in these studies is the high initial stress associated with participating in extreme sports. Men with lower self-confidence and higher levels of stress or anxiety may find it challenging to engage in these activities (15). Furthermore, social anxiety disorders often co-occur with depression, which can diminish initial interest in such activities (16). More importantly, extreme sports carry significant risks and threats, which may lead to injury, especially for beginners or individuals with social anxiety disorders (17). While extreme sports may foster emotional growth and psychological resilience through high-stress exposure, the intensity and unpredictability of these activities in real-world settings can be overwhelming—especially for individuals with social anxiety disorder (SAD). In contrast, controlled exposure to stressors within virtual reality provides a more manageable and safe environment that allows individuals to gradually build resilience without facing the potentially harmful consequences of real-world participation (18). For this reason, real-world environments may not be suitable for such individuals due to these risks. In contrast, virtual reality (VR), as an advanced technology, offers simulated environments where individuals can practice and face anxiety-inducing situations within controlled settings without the need for direct exposure to social situations (19). For example, Recent studies have demonstrated that immersive VR simulations—especially those replicating high-adrenaline activities—can elicit physiological arousal, including increased heart rate, cortisol levels, and adrenaline release, closely mimicking real-world responses (20, 21). Moreover, VR environments have been shown to enhance users’ perceived self-efficacy through safe yet realistic exposure to challenging tasks (22). These findings suggest that VR-based extreme sports can replicate not only the sensory and cognitive stressors of real-life experiences but also foster psychological growth, resilience, and emotion regulation in individuals with social anxiety disorders. Thus, this study aims to create a safe yet thrilling experience for men with social anxiety disorder by employing extreme sports simulations in VR. It seeks to reveal the effects of VR as a safe, non-pharmacological intervention. In recent years, there has been a growing interest in using technology-based approaches to address various behavioral and emotional disorders, one of which is VR. This technology has shown promise as an innovative and effective tool for confronting various social situations (23). VR allows for the creation of simulated environments, enabling individuals to practice and engage with anxiety-inducing scenarios in a controlled setting without real-world exposure (24).

This study, therefore, seeks to conduct a quasi-experimental investigation focused on men with social anxiety disorder to assess the effectiveness of extreme sports games in a virtual reality environment. It aims to uncover the potential effects of this intervention on levels of anxiety, stress, and depression in men. Using reliable measurement tools, researchers hope to precisely evaluate both the positive and negative impacts of these sports experiences on participants’ anxiety and stress levels. The results of this study may prove highly valuable in therapeutic contexts as a non-pharmacological and counseling intervention, offering innovative strategies to improve the psychological well-being of affected men. Given advancements in VR technology, simultaneously addressing social issues and alleviating negative emotions may represent a significant step toward developing new therapeutic approaches and could pave the way for the application of VR in treating other behavioral and medical disorders.

## 2 Literature review

### 2.1 Social anxiety disorder in men

Social Anxiety Disorder (SAD) is characterized by an intense fear of social situations where one might be negatively evaluated. It is among the most common anxiety disorders, with lifetime prevalence estimates up to ~12% in the general population (2). While women have a slightly higher incidence of SAD, men with social anxiety represent a substantial and important subgroup. Notably, men and women appear equally likely to seek treatment, but men may present with less severe symptoms, possibly reflecting gender differences in help-seeking thresholds. These differences are often rooted in masculine norms. Traditional masculinity emphasizes stoicism, self-reliance, and fearlessness, which can make the open acknowledgment of anxiety particularly challenging for men. Indeed, men experiencing anxiety often prefer to rely on problem-solving and independent coping strategies rather than reaching out for professional help (25). This tendency can lead to underreporting of symptoms and delays in treatment. Men may conceal or internalize feelings of fear and worry to avoid the shame or “weakness” associated with anxiety in the context of masculine expectations (5, 26).

Qualitative research provides insight into how men cope with social anxiety in their daily lives. In a recent study exploring mental health strategies among men with anxiety, the home environment emerged as a double-edged sword: it was described as a private refuge where men felt safe to hide from social fears, yet it could also become a place of despair and isolation (27). Men in similar contexts have reported using the home as a space to “conceal and deal with” their mental health challenges on their own terms. However, the same four walls that offered protection from judgment also sometimes left them feeling trapped and alone, potentially exacerbating feelings of hopelessness (15). These findings underscore the importance of developing interventions that respect men’s preference for privacy and autonomy while also breaking the cycle of isolation. In other words, supporting men with SAD may require innovative approaches that engage them in a comfortable setting (even their home) without sacrificing the therapeutic benefits of social exposure and interaction (28, 29).

### 2.2 Alternative approaches: physical activity and VR

As a result of treatment avoidance, non-pharmacological interventions like exercise and Virtual Reality Exposure Therapy (VRET) have gained traction. Regular physical activity has demonstrated anxiolytic effects, with studies showing reductions in anxiety symptoms and improvements in mood, particularly among adult men (30). Extreme sports, which involve controlled exposure to high-stress environments, have been associated with enhanced emotional regulation and resilience. These sports may be especially appealing to men due to their alignment with traditional masculine norms like fearlessness and control. However, engaging in extreme sports poses accessibility and safety concerns (7). Many individuals may lack time, resources, or physical capability to participate in high-risk activities such as rock climbing or skydiving. The American Psychological Association has acknowledged these risks and emphasized the need for structured, safer alternatives. This limitation presents a clear research gap: while the benefits of extreme sports on anxiety reduction are evident, their real-world implementation is impractical or unsafe for many.

Virtual Reality (VR) provides a potential solution. VRET simulates anxiety-provoking scenarios within a controlled environment, allowing users to practice coping strategies safely. Meta-analyses show that VRET is comparable in effectiveness to in-person CBT (19, 31). Additionally, studies highlight the suitability of VR for men who prefer privacy and action-based interventions (32). Despite minor technical barriers like simulator sickness or realism issues, advancements in VR technology have improved user experience and reduced attrition.

### 2.3 Research gap: extreme sports exergaming in VR

While VR and physical activity each offer significant therapeutic value, few studies have explored their integration. Specifically, there is limited research examining whether extreme sports exergaming in VR can replicate the mental health benefits of real-life extreme sports—without the associated dangers. This represents a notable research gap. Extreme sports in VR could offer the arousal, challenge, and self-efficacy benefits seen in physical participation, while remaining accessible, affordable, and safe. Therefore, the present study aims to investigate the potential of VR-based extreme sports exergaming as a novel non-pharmacological intervention for men with SAD. By combining the emotional engagement of extreme sports with the accessibility of VR, this approach seeks to bridge a crucial gap in existing therapeutic options—particularly for individuals who are unable or unwilling to engage in real-world physical alternatives.

## 3 Methodology

### 3.1 Participants

Participants were recruited from public psychology clinics, university counseling centers, and mental health associations in Beijing, China, where psychological services for individuals with anxiety-related conditions are commonly offered. Recruitment efforts included printed flyers, digital advertisements, and referrals by licensed therapists working in these institutions. Initial outreach resulted in 98 adult male volunteers who expressed interest in participating. All potential participants underwent a structured clinical interview based on DSM-5 criteria, conducted by licensed clinical psychologists. Diagnoses were confirmed solely by clinical evaluation, ensuring each participant met the diagnostic threshold for social anxiety disorder (SAD).

After excluding individuals undergoing active intensive therapy (*n* = 8), presenting with comorbid psychiatric disorders (*n* = 4), or withdrawing prior to formal enrollment (*n* = 2), a total of 84 participants completed the eligibility process and were enrolled in the study. Participants were then randomly assigned to either the experimental group (*n* = 42) or the control group (*n* = 42). Assignment was stratified based on age (±3 years) and general physical health (self-reported ability to engage in moderate physical activity). Inclusion criteria required participants to be adult males aged under 25 to 40 years, with a verified diagnosis of SAD, no severe physical limitations, and no recent changes in pharmacological or psychological treatment within the past 3 months. Table 1 provides detailed demographic information, including age, marital status, and the duration since diagnosis, for each participant in the study.

**Table 1:** Demographic information of the participants.

Variable	Type	Frequency	Percentage
Gender	Men	84	100%
Marital status	Single	58	69/0%
	Married	26	30/9%
Age	Under 25	9	17/8%
	Between 25 and 30 years	22	26/1%
	Between 31 and 35 years	28	33/3%
	Between 36 and 40 years	25	19/0%
Duration of disorder diagnosis	Between 1 and 2 years	22	26/1%
	Between 3 and 5 years	41	48/8%
	Between 6 and 8 years	21	25/0%

### 3.2 Study design

This study employed a quasi-experimental design, dividing participants into experimental and control groups. The experimental group engaged in extreme sports games within a virtual reality (VR) environment, while the control group participated in conventional physical activities such as running and futsal without VR intervention. A pre-test and post-test model was utilized to assess changes in levels of anxiety, depression, and stress among the participants. This design enabled a comparative analysis of the effects of VR-based extreme sports games on anxiety, depression, and stress levels, as opposed to traditional sports activities.

### 3.3 Methodology

After recruiting participants, baseline levels of anxiety, depression, and stress were assessed in both groups using the standardized Depression, Anxiety, and Stress Scale (DASS-21) questionnaire. The experimental group then participated in extreme sports games within a virtual reality (VR) environment, while the control group engaged in traditional physical exercises. This group functioned as an active control, participating in physical activities of comparable intensity, frequency, and duration to those of the experimental group, but without exposure to virtual reality. To manage potential confounding effects, both groups followed identical weekly schedules (three 45–60-min sessions per week over 4 weeks), received similar instructions, and were supervised by the same type of staff (one sports expert and one facilitator). All sessions were conducted in similar indoor settings to ensure consistency across environmental conditions.

Participation was voluntary, and no fees were charged for the sessions as an acknowledgment of participants’ commitment. Upon completion of the training sessions for both the experimental and control groups, participants completed the DASS-21 questionnaire again in a post-test format to measure levels of anxiety, depression, and stress.

### 3.4 Measurement tools

The assessment tool used in this study was the Depression, Anxiety, and Stress Scale (DASS-21), initially developed by Lovibond (45). The questionnaire was modified to align with the study’s target population, and, based on feedback from qualified psychologists, one additional question was added to the anxiety section to fully address the research objectives. Reliability for each section of the questionnaire was determined using Cronbach’s alpha, yielding coefficients of 0.97, 0.92, and 0.95, indicating high reliability for the tool (33). The questionnaire consisted of 27 questions in total: five questions gathered demographic and qualitative data, while the remaining 22 questions assessed key areas—stress (7 items), depression (7 items), and anxiety (8 items).

### 3.5 Statistical analysis

The Kolmogorov–Smirnov test was used to examine the normality of the variables, with *p*-values exceeding 0.05 for all components, indicating normal distribution. Levene’s test was applied to assess the homogeneity of variances between the experimental and control groups, revealing a violation of this assumption (*p* < 0.05). Consequently, Analysis of Covariance (ANCOVA) was employed to control for baseline differences (34). Separate univariate ANOVAs were not conducted to prevent redundancy and contradictory results, given that ANCOVA adjusts for baseline disparities. Effect sizes were calculated using partial eta squared, with Cohen’s (44) criteria for interpretation: small (0.01), medium (0.06), and large (0.14). To minimize Type I error, a Bonferroni correction was applied, specifically for multiple comparisons across the three dimensions of anxiety, depression, and stress, with significance set at *p* < 0.05 for all tests (35). Analyses were conducted using SPSS version 26.

### 3.6 Implementation of VR-based extreme sports games

Guidance from three VR experts and researchers in this field was sought to implement the extreme sports games in a virtual reality setting. Initially, an in-person meeting was held with participants to explain the entire research process, including the voluntary nature of participation, and written consent was obtained from all individuals. A structured 12-session program was then established, consisting of three sessions per week over a four-week period, with each session lasting approximately 45 to 60 min. Classes were provided at no cost, along with sportswear and shoes to encourage participation and offset any inconvenience caused by the study requirements.

During the intervention for the experimental group, participants engaged in extreme sports games using Meta Quest VR headsets, specifically the third-generation model with a 512GB capacity. Throughout the VR training sessions, a game expert and a sports expert were present to guide and support participants. The VR experience consisted of immersive simulations of high-adrenaline activities such as indoor rock climbing, wingsuit flying, and virtual skydiving. These simulations featured high-resolution 360-degree visual environments, spatial audio, and responsive feedback through handheld controllers. Visual realism was enhanced using photorealistic textures and natural motion tracking, enabling participants to move and interact freely in the virtual space. The design emphasized sensory immersion and presence, allowing users to feel physically and emotionally engaged. Sessions were conducted individually in quiet rooms with minimal distractions to further increase immersion and comfort.

## 4 Results

As observed in Table 2, all research variables possess a normal distribution according to the Kolmogorov–Smirnov test, as the obtained values indicate (*c* = Significance Correction, *d* = lower bound of the true significance). Therefore, with 95% confidence, it can be claimed that all research variables follow a normal distribution; hence, parametric tests are used for analyzing the research hypotheses (Tables 3, 4).

**Table 2:** Kolmogorov–Smirnov test.

Group	Control		Experiment	
	Test statistic	Significance level	Test statistic	Significance level
Pre-test- depression	0.130	0.070c	0.083	0.200c,d
Pre-test- anxiety	0.095	0.200c,d	0.131	0.068c
Pre-test- stress	0.127	0.088c	0.150	0.018c
Post-test- depression	0.122	0.118c	0.123	0.111c
Post-test- anxiety	0.176	0.002c	0.169	0.004c
Post-test- stress	0.193	0.000c	0.271	0.000c

(c = Significance Correction, d = lower bound of the true significance).

**Table 3:** Presents key descriptive indicators pertinent to the research.

Variable	Group		*N*	Mean	Std. deviation
Control	Pre-test	Depression	42	45.7619	3.59410
		Anxiety	42	31.6905	3.40364
		Stress	42	20.9524	2.49832
	Post-test	Depression	42	43.0952	3.76641
		Anxiety	42	30.2381	2.90340
		Stress	42	19.5238	2.22226
Experimental	Pre-test	Depression	42	45.8571	3.55151
		Anxiety	42	31.7143	3.22555
		Stress	42	21.3095	2.65484
	Post-test	Depression	42	25.5714	2.52927
		Anxiety	42	17.8810	2.25456
		Stress	42	11.4048	2.13061

**Table 4:** The results of Levene’s test.

Variable	*F*	df1	df2	Sig.
Depression	0.161	1	82	0.689
Anxiety	0.207	1	82	0.650
Stress	0.877	1	82	0.352

### 4.1 Levene’s test

As observed, the assumption of equal variances holds for all research variables (*p* > 0.05). Given this and the fulfillment of all other preliminary assumptions, the data in this study are suitable for entry into covariance analysis (ANCOVA). This allows for a reliable examination of the differences between the two groups in the dependent variables.

Based on the data presented in Table 5, a significant effect of the VR-based extreme sports intervention on depression levels was identified. The Type III Sum of Squares for depression was 6481.671, with an *F* value of 879.424 (*p* < 0.001), and a partial eta squared (*η*^2^) of 0.916, indicating that the intervention accounted for 91.6% of the variance in depression levels among men with social anxiety disorder. Additionally, a significant difference in the adjusted mean depression scores between the control and experimental groups was observed (Type III Sum of Squares = 246.905, *F* = 33.500, *p* < 0.001), with a partial eta squared of 0.293, highlighting the intervention’s substantial impact.

**Table 5:** The results of covariance analysis.

Source	Type III sum of squares	df	Mean square	*F*	Sig.	Partial eta squared
Depression						
Pre	246.905	1	246.905	33.500	0.000	0.293
Group	6481.671	1	6481.671	879.424	0.000	0.916
Error	596.999	81	7.370			
Anxiety						
Pre	200.979	1	200.979	46.111	0.000	0.363
Group	3212.473	1	3212.473	737.046	0.000	0.901
Error	353.045	81	4.359			
Stress						
Pre	94.391	1	94.391	25.988	0.000	0.243
Group	1428.428	1	1428.428	393.273	0.000	0.829
Error	294.204	81	3.632			

According to the results in Table 5, after controlling for the pre-test variable, the computed F statistic indicates a significant difference in the adjusted mean anxiety scores between the control and experimental groups in the post-test phase (*p* < 0.05). This finding leads to the rejection of the null hypothesis, confirming that VR-based extreme sports gaming has a significant effect on the anxiety levels of men with social anxiety disorder. The partial eta squared value was 0.901, showing that 90% of the variance in anxiety levels among men with social anxiety disorder was accounted for by the VR-based extreme sports intervention. Also, after adjusting for pre-test variables, the F statistic shows a significant difference in stress scores between the control and experimental groups at post-test (*p* < 0.05). This finding supports rejecting the null hypothesis, suggesting that VR-based extreme sports gaming significantly impacts stress levels in men with social anxiety disorder. The effect size (*η*^2^ = 0.829) suggests that 82% of the variance in stress levels is explained by the intervention.

**Figure 1:**
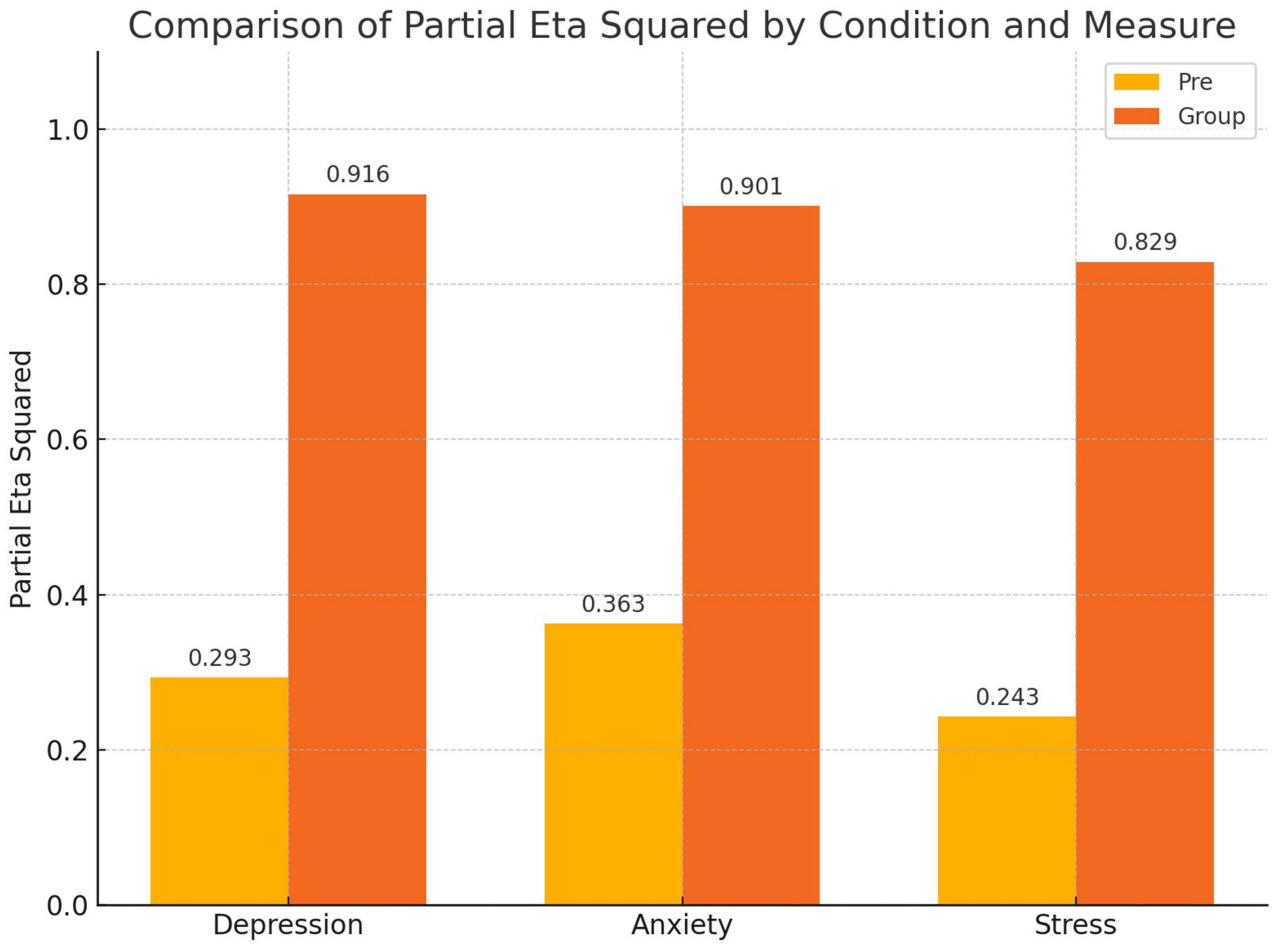
The differences between the pre-test and post-test.

Figure 1 illustrates the differences between the pre-test and post-test results to facilitate a clearer understanding of the changes.

## 5 Discussion

Men with social anxiety disorder (SAD) often experience intense and persistent fear in social situations, typically due to fear of negative judgment, low self-confidence, and heightened stress in social settings. This disorder can significantly affect men’s social functioning, occupational performance, and overall mental health, impeding everyday activities such as interpersonal and workplace interactions, and potentially leading to comorbid psychological conditions over time (2). Many men, due to certain personality traits or concerns about side effects, may refuse pharmacological treatment altogether (5). In response, the present study explored a non-pharmacological intervention using extreme sports activities delivered within a virtual reality (VR) environment for men with SAD.

The findings suggest that VR-based extreme sports games may effectively reduce stress levels in this population. These results align with previous research demonstrating the stress-reducing potential of VR sports games. For example, one study found that participants who engaged in VR sports games over a six-week period reported significant reductions in stress and anxiety, attributed to the sense of presence and engagement provided by the virtual environment, which helped shift attention away from negative thoughts and social concerns (36). However, not all studies have found consistent results. For instance, Wechsler and colleagues reported that some patients preferred in-person therapy or real-life exposure, citing the artificial nature of VR environments and the absence of human interaction as limiting factors (32). This inconsistency may be due to earlier technological limitations that hindered immersive realism. In recent years, however, virtual reality has advanced significantly, offering more naturalistic and immersive experiences (37). Recent reviews, such as that by Meshkat et al. support the effectiveness of VR-based sports games in stress reduction (18), highlighting the value of safe and controlled virtual settings. These environments may enhance participants’ sense of safety and comfort, which in turn can empower individuals to gradually manage their stress and fears, potentially preparing them for real-world situations over time.

The findings of the present study revealed that VR-based extreme sports games can support emotional regulation by significantly reducing individuals’ arousal levels (*p* < 0.05). These results are consistent with previous research suggesting that extreme sports games, due to their physical stimulation and high attentional demand, can elicit elevated levels of excitement without exposing participants to the risks inherent in real-world environments. Such emotional regulation can foster positive emotional experiences and diminish negative affect, which is particularly relevant for men with social anxiety disorder (38). For instance, Rose et al. noted that participating in moderate-to-intense VR sports activities helped reduce negative emotions while enhancing positive emotional states (28). Similarly, Pallavicini et al. reported that VR-based games may contribute to reduced anxiety and improved emotional regulation following gameplay (39). In line with these findings, Pustovojt demonstrated that extreme sports impact not only physiological responses but also psychological resilience, with athletes showing enhanced emotional control and stress management in real-world scenarios (9). In the current study, it was also observed that male participants engaged in VR extreme sports expressed intense emotions freely, without experiencing embarrassment or fear, which likely facilitated emotional release. With continued participation, VR-based extreme sports may help men with SAD gradually develop greater emotional regulation and resilience over time.

The findings of the present study also indicated a reduction in anxiety levels among men. In fact, simulated exposure to social situations in a virtual reality (VR) environment may contribute to alleviating social anxiety (29). Related studies on Virtual Reality Exposure Therapy (VRET) have shown significant reductions in social anxiety levels. For example, in a systematic review of VRET for social anxiety, researchers reported that this approach effectively reduced social anxiety symptoms, especially for individuals unable to use in-person exposure methods (40). Our findings align with previous studies by Chung on the use of VR extreme sports as an effective method for reducing social anxiety. Another systematic review assessing VR’s effectiveness in social anxiety treatment demonstrated that simulated VR exposure could significantly alleviate social anxiety symptoms (29). Additional research indicated that VR-based exposure therapy could be as effective as in-person exposure, providing users with a controlled environment that helps reduce anxiety and enhance social interactions (41). However, Some studies have reported conflicting findings, suggesting that virtual reality games may actually increase anxiety and arousal in certain users. For instance, research has found that VR environments can lead to heightened anxiety, especially when the realism of simulated interactions is limited or when the games involve intense or competitive scenarios. These factors can contribute to an escalation in users’ stress and arousal levels, potentially counteracting the intended calming effects (42). This effect may be attributed to the participants engaging in VR-based combat or conflict scenarios, which can increase anxiety. In contrast, high-adrenaline sports games often provide positive, manageable challenges that are less likely to induce anxiety. Individuals prone to anxiety may benefit from controlled positive challenges that elevate adrenaline in a manageable way, enhancing feelings of success and capability—a benefit often absent in combat or conflict-based VR games (43). Further supporting this view, Sheng Yen Lee found that men engaging in VR extreme sports, such as rock climbing and high-altitude activities, often experience improved self-confidence and reduced anxiety (11). Other studies, such as Makar et al. (12), Lin Wang et al. (13), and Narelle Eather (14), highlighted the positive impact of sports activities, including extreme VR games, on reducing daily psychological stress and improving stress and anxiety levels in individuals’ day-to-day lives, which is consistent with the present study’s results.

Overall, the findings of this study indicate that VR-based extreme sports games can serve as an effective, non-pharmaceutical tool for reducing stress and anxiety, while enhancing positive emotions in men with social anxiety disorder. In this context, VR not only supports emotional regulation and self-confidence building, but also offers a motivating and safe alternative for individuals who may be reluctant to engage in traditional therapies. The realistic simulation of high-adrenaline environments enables users to experience the psychological benefits of extreme sports without exposure to the physical risks inherent in real-world settings. However, it is important to consider that individual differences—such as prior gaming experience, affinity for technology, or susceptibility to VR-induced discomfort (e.g., motion sickness)—may influence how participants engage with and respond to the intervention. Recognizing such variability is essential for interpreting the results and for guiding the development of more tailored and inclusive VR-based therapeutic strategies in future research.

### 5.1 Limitations

This study has several limitations that should be acknowledged. First, the experimental design involved two simultaneous variables within the intervention group: the use of immersive virtual reality (VR) technology and participation in extreme sports simulations. In contrast, the control group engaged in conventional physical activities without VR. As a result, it is not possible to determine whether the observed improvements in anxiety, stress, and depression were driven by the VR medium itself, the intense and novel nature of extreme sports, or the interaction of both. Thus, Future studies are encouraged to include an additional comparison group—such as participants exposed to VR-based non-extreme sports—to better disentangle the individual contributions of sport intensity, technological immersion, and novelty effects.

Another limitation of this study is the possibility of novelty effects associated with the use of virtual reality (VR). For participants with no prior VR experience, the immersive and technologically advanced nature of the platform may have elicited heightened excitement or engagement, which could lead to temporary improvements in mood and anxiety symptoms. This raises the possibility that some of the observed benefits may be due to the initial novelty of the VR environment rather than its sustained therapeutic value. To address this concern, the study employed a pretest-posttest design to capture changes over the course of the intervention. However, future research should incorporate longer-term follow-up assessments to determine whether the psychological benefits observed are sustained over time and to distinguish novelty-driven effects from enduring therapeutic outcomes. Additionally, the relatively small sample size may limit the generalizability of the findings, and future studies with larger and more diverse samples are recommended.

## 6 Conclusion

The present study’s findings reveal that virtual reality-based extreme sports games can effectively serve as a safe and non-pharmacological intervention for reducing symptoms of depression, anxiety, and stress in men diagnosed with social anxiety disorder. These outcomes underscore the value of incorporating virtual reality as an innovative adjunct in psychological interventions, offering a promising option for individuals hesitant to pursue conventional therapeutic or pharmacological treatments. The marked improvements observed suggest that virtual reality interventions may play a significant role in enhancing mental health outcomes for this population. Future research is warranted to examine the long-term effects of this approach, assess the durability of symptom relief, and further investigate the mechanisms by which virtual reality can support anxiety disorder interventions.

## Data Availability

The raw data supporting the conclusions of this article will be made available by the authors, without undue reservation.
